# Early initiation of low-dose corticosteroid therapy in the management of septic shock: a retrospective observational study

**DOI:** 10.1186/cc10601

**Published:** 2012-01-07

**Authors:** Hye Yun Park, Gee Young Suh, Jae-Uk Song, Hongseok Yoo, Ik Joon Jo, Tae Gun Shin, So Yeon Lim, Sookyoung Woo, Kyeongman Jeon

**Affiliations:** 1 Division of Pulmonary and Critical Care Medicine, Department of Medicine, Samsung Medical Center, Sungkyunkwan University School of Medicine, 81 Irwon-ro, Gangnam-gu, Seoul, 135-710, Republic of Korea; 2 Department of Emergency Medicine, Samsung Medical Center, Sungkyunkwan University School of Medicine, 81 Irwon-ro, Gangam-gu, Seoul, 135-710, Republic of Korea; 3 Biostatistics Team, Samsung Biomedical Research Institute, 81 Irwon-ro, Gangnam-gu, Seoul, 135-710, Republic of Korea

## Abstract

**Introduction:**

The use of low-dose steroid therapy in the management of septic shock has been extensively studied. However, the association between the timing of low-dose steroid therapy and the outcome has not been evaluated. Therefore, we evaluated whether early initiation of low-dose steroid therapy is associated with mortality in patients with septic shock.

**Methods:**

We retrospectively analyzed the clinical data of 178 patients who received low-dose corticosteroid therapy for septic shock between January 2008 and December 2009. Time-dependent Cox regression models were used to adjust for potential confounding factors in the association between the time to initiation of low-dose corticosteroid therapy and in-hospital mortality.

**Results:**

The study population consisted of 107 men and 71 women with a median age of 66 (interquartile range, 54 to 71) years. The 28-day mortality was 44% and low-dose corticosteroid therapy was initiated within a median of 8.5 (3.8 to 19.1) hours after onset of septic shock-related hypotension. Median time to initiation of low-dose corticosteroid therapy was significantly shorter in survivors than in non-survivors (6.5 hours versus 10.4 hours; *P *= 0.0135). The mortality rates increased significantly with increasing quintiles of time to initiation of low-dose corticosteroid therapy (*P *= 0.0107 for trend). Other factors associated with 28-day mortality were higher Simplified Acute Physiology Score (SAPS) 3 (*P *< 0.0001) and Sequential Organ Failure Assessment (SOFA) scores (*P *= 0.0007), dose of vasopressor at the time of initiation of low-dose corticosteroid therapy (*P *< 0.0001), need for mechanical ventilation (*P *= 0.0001) and renal replacement therapy (*P *< 0.0001), while the impaired adrenal reserve did not affect 28-day mortality (81% versus 82%; *P *= 0.8679). After adjusting for potential confounding factors, the time to initiation of low-dose corticosteroid therapy was still significantly associated with 28-day mortality (adjusted odds ratio (OR) 1.025, 95% confidence interval (CI) 1.007 to 1.044, *P *= 0.0075). The early therapy group (administered within 6 hours after the onset of septic shock, *n *= 66) had a 37% lower mortality rate than the late therapy group (administered more than 6 hours after the onset of septic shock, *n *= 112) (32% versus 51%, *P *= 0.0132).

**Conclusions:**

Early initiation of low-dose corticosteroid therapy was significantly associated with decreased mortality.

## Introduction

Septic shock is one of the most common causes of death in patients admitted to the ICU [1,2]. Despite advances in early and appropriate antibiotic therapy, initial resuscitation, source control, and organ support, septic shock remains a major source of short-term and long-term morbidity and mortality [3]. Although multiple adjunctive therapies for septic shock have been developed and studied in clinical trials, few of these methods have demonstrated improvements in survival [4]. Corticosteroid treatment has been studied extensively as adjunctive therapy in patients with septic shock for over 40 years. Initial trials investigating a short course of high-dose corticosteroid as an anti-inflammatory regimen found no evidence of a survival benefit [5,6]. In contrast, more recent trials demonstrated that a longer course (≥ 5 days) of low-dose corticosteroid resulted in shock reversal [7] and improved mortality rate [8,9]. Based primarily on a large randomized, multicenter, controlled study performed by Annane *et al*. [9], the Surviving Sepsis Campaign guidelines recommend the use of low-dose corticosteroid in patients with septic shock requiring vasopressors despite fluid replacement [3]. Recently, however, the Corticosteroid Therapy of Septic Shock (CORTICUS) study by Sprung *et al*. [10] did not demonstrate an improvement in mortality rate after low-dose corticosteroid therapy in patients with septic shock. The contradictory findings of these two large multicenter studies generated controversy regarding the appropriateness of corticosteroid therapy in patients with septic shock.

Although a number of factors may have accounted for the differences in the results of these two large multicenter studies, the difference in the time window of enrollment should be considered [11]. Annane *et al*. initially enrolled patients in the study within three hours of the onset of shock, and this time window from the onset of septic shock to randomization was increased to eight hours [9]. In contrast, in the CORTICUS study, patients were required to undergo randomization within 24 hours after the onset of septic shock, and this time window was subsequently increased to 72 hours [10]. Therefore, it is probable that the benefit of low-dose corticosteroid therapy may diminish with a delay in instituting the treatment [12]. However, there have been no reports regarding the association between the timing of corticosteroid therapy and mortality in patients with septic shock. Therefore, we performed a retrospective analysis of the clinical data from patients who received low-dose corticosteroid therapy for septic shock to determine whether early initiation of this therapy is associated with decreased mortality in such cases.

## Materials and methods

This was a retrospective study performed in a cohort of patients with septic shock admitted to the medical ICU of Samsung Medical Center (a 1,960-bed, university-affiliated, tertiary referral hospital in Seoul, South Korea), which has 30 beds providing care for approximately 860 critically ill patients per year. The study was approved by the institutional review board of Samsung Medical Center to review and publish information from the patients' records. Informed consent was waived because of the retrospective nature of the study.

### Study population

All consecutive patients with septic shock admitted to the medical ICU between January 2008 and December 2009 were screened for inclusion in this study if they had received low-dose corticosteroid therapy (≤ 300 mg/day of hydrocortisone or equivalent) for septic shock. Patients were excluded if they were less than 18 years old, had received systemic corticosteroid therapy within the last 3 months before septic shock, had received high-dose steroid therapy (> 300 mg/day of hydrocortisone or equivalent), or did not receive low-dose corticosteroid therapy for septic shock. Additionally, immunocompromised patients, such as those with HIV infection and those who had undergone stem cell or solid organ transplantations, were excluded from the study.

### Initial resuscitation and hemodynamic management for septic shock

A specific protocol for early recognition and management of patients with severe sepsis or septic shock has been implemented in our center since 2004 [13]. Additionally, to improve the compliance with the initial resuscitation bundle and management for sepsis, we revised, approved, and promoted our early goal-directed therapy (EGDT) protocol with an educational program named 'Emergency Approach to Sepsis Treatment (EAST)' in early 2008. Our EGDT protocol is an adaptation of the protocol reported by Rivers *et al*. [14]. Fluid resuscitation and hemodynamic monitoring were initiated in patients fulfilling the criteria for severe sepsis or septic shock, with placement of a central venous catheter *via *the internal jugular or subclavian vein approach for central venous pressure (CVP) and central venous oxygen saturation (ScvO_2_) monitoring. Broad-spectrum antibiotics were administered as soon as possible. Hemodynamic resuscitation was conducted according to a predetermined treatment plan. First, isotonic crystalloid was administered in boluses to target CVP ≥ 8 mmHg. Second, systolic blood pressure ≥ 90 mmHg or mean arterial pressure (MAP) ≥ 65 mmHg, if not achieved with fluid administration, was targeted by initiating and titrating vasopressors (preferably norepinephrine as a first-line agent) to achieve this desired blood pressure. Finally, ScvO_2 _≥ 70% was targeted after CVP and blood pressure goals were met. If ScvO_2 _was lower than 70% and the hematocrit was lower than 30%, packed red blood cells were transfused to achieve a hematocrit of at least 30%. If ScvO_2 _remained lower than 70% when hematocrit was 30% or higher, dobutamine was initiated at the treating physician's discretion and titrated in attempts to reach ScvO_2 _≥ 70%. When the patient remained hypotensive after at least one hour of resuscitation with fluids and vasopressor [9], low-dose corticosteroid therapy was recommended as soon as possible after sampling for adrenocorticotropic hormone (ACTH) test if possible. However, the time to initiation of low-dose corticosteroid therapy was decided by the treating physician in the emergency department or ICU. Hydrocortisone was administered intravenously every 6 hours as a 50-mg bolus for 5 days and then tapered (50 mg intravenously every 12 hours for 3 days, followed by 50 mg intravenously daily for 3 days). Fludrocortisone was not to be administered in conjunction with hydrocortisone. If hemodynamic stabilization was achieved, the vasopressor was tapered based on the decision of the attending physician, keeping MAP above 65 mmHg and urinary output higher than 0.5 mL/kg/hour.

### Definitions

Septic shock was defined as sepsis with acute circulatory failure characterized by persistent arterial hypotension (systolic arterial pressure < 90 mmHg in 156 patients (88%), mean arterial pressure < 60 mmHg in 147 (83%)], or a reduction in systolic blood pressure > 40 mmHg from baseline in 21 (12%)) despite adequate volume resuscitation [15]. Organ dysfunction was defined as Sequential Organ Failure Assessment (SOFA) score ≥ 2 for each organ system [15,16]. Critical-illness-related corticosteroid insufficiency (CIRCI) was diagnosed by a delta serum cortisol level of < 9 μg/dL after ACTH (250 μg) administration (relative adrenal insufficiency) or random total serum cortisol < 10 μg/dL [12]. Appropriate antibiotic therapy was considered if the initially prescribed antibiotics were active against the identified pathogens, based on *in vitro *susceptibility testing. The time to initiation of antibiotic therapy relative to the onset of septic shock was defined as the time from the initial onset of septic shock-related hypotension to the initiation of antibiotics [17]. Time to initiation of low-dose corticosteroid therapy was defined as the time from the initial onset of septic shock-related hypotension to the initiation of corticosteroid. Reversal of shock was defined as the maintenance of a systolic blood pressure of at least 90 mmHg without vasopressor support for at least 24 hours [8,10].

### Data collection

The following data recorded at the time of initiation of low-dose corticosteroid therapy were collected from the electronic medical records: general characteristics of the patients including demographic data, infection source, laboratory measurements including initial lactate, amount of fluid administered before initiation of vasopressor, doses of vasopressor (norepinephrine or equivalent), and organ dysfunction. The severity of illness was assessed by Simplified Acute Physiology Score 3 (SAPS 3) [18] and SOFA scores [19]. SAPS 3 was calculated as the worst value for that variable during the first one hour of ICU admission, and SOFA scores were calculated from the data on admission. On ICU admission, the following conditions were evaluated: the need for mechanical ventilation and renal replacement therapy, appropriateness of empirical antibiotic therapy, and presence of bacteremia.

The primary outcome was 28-day mortality. To address the primary research question of whether mortality and time to initiation of low-dose steroid therapy are associated, we considered age, gender, time to initiation of antibiotic therapy and severity of illness as potential confounders [17,20]. The secondary outcomes were reversal of shock, ICU mortality, in-hospital mortality and the duration of ICU and hospital stay.

### Statistical analysis

Data are presented as medians and interquartile range (IQR) for continuous variables and as numbers (percentages) for categorical variables. Data were compared using the Mann-Whitney *U*-test for continuous variables and the χ^2 ^or Fisher's exact test for categorical variables. To assess whether there was an association between 28-day mortality and the time to initiation of low-dose corticosteroid therapy, the Mantel-Haenszel test was used to examine trends across the quintiles of time.

A logistic regression model was used to adjust for potential confounding factors in the association between the time to initiation of low-dose corticosteroid therapy and 28-day mortality. Three models were constructed: model 1 was adjusted for age and gender, model 2 was additionally adjusted for time to initiation of antibiotic therapy and severity of illness, and model 3 additionally included factors with *P *< 0.25 in univariate analysis as confounding factors [21]. Data are presented as odds ratios (OR) with 95% confidence intervals (CI).

The baseline characteristics and outcome measures of interest were then compared between the patients receiving low-dose corticosteroid therapy within versus after six hours from the initial onset of septic shock-related hypotension. Kaplan-Meier estimation was used to determine the 90-day survival curves for these two times to initiation of low-dose corticosteroid therapy, which were then compared using the log-rank test for survival data. Statistical analyses were performed using SAS version 9.1 (SAS Institute, Cary, NC), and two-sided *P *< 0.05 was considered significant.

## Results

Over the study period, a total of 300 patients with septic shock were admitted to the medical ICU. Of these, 112 patients were excluded according to the exclusion criteria: systemic corticosteroid therapy within the last 3 months before septic shock (*n *= 63), high-dose steroid therapy (*n *= 40), and immunocompromised status (*n *= 9). Moreover, ten patients who were transferred from other hospitals after initiation of vasopressors were also excluded. Finally, 178 patients who received low-dose steroid therapy for septic shock were included in this study.

The baseline characteristics of the patients at the time of initiation of low-dose corticosteroid therapy are presented in Table 1. There were 107 male and 71 female patients, and the median age was 66 (IQR, 54 to 71) years. The median SAPS 3 and SOFA scores on ICU admission were 81 (72 to 90) and 11 (9 to 13), respectively. Pneumonia was the most common cause of septic shock (*n *= 80, 45%), followed by gastrointestinal tract infection (*n *= 47, 26%) and urinary tract infection (*n *= 19, 11%). Microbiologically proven infection was observed in 58% of the cohort, and 37% had proven bloodstream infection. Seventy-nine percent of these patients received appropriate antibiotic treatment. Mechanical ventilation was needed in 124 (70%) patients, and renal replacement therapy was needed in 58 (33%) patients on ICU admission. Median dose of vasopressor (norepinephrine or equivalent) at the time of initiation of low-dose corticosteroid therapy was 0.48 μg/kg/minute. ACTH stimulation tests were performed in 96 (54%) patients, and CIRCI was diagnosed in 78 patients. The number of patients with relative adrenal insufficiency defined by a delta serum cortisol level of < 9 μg/dL after ACTH administration was 71 (91% of patients with CIRCI). The median time to initiation of low-dose corticosteroid therapy was 8.5 (3.8 to 19.1) hours.

**Table 1:** Baseline characteristics of 178 patients with septic shock receiving low-dose corticosteroid therapy

Characteristics	Number (%) or median (interquartile range)
Age, years	66 (54-71)
Sex, male	107 (60)
Severity of illness	
SAPS 3	81 (72-90)
SOFA	11 (9-13)
Site of infection	
Lung	80 (45)
Gastrointestinal tract	47 (26)
Urinary tract	19 (11)
Catheter related	5 (3)
Skin and soft tissue	6 (3)
Others	21 (12)
Acquisition of infection	
Community	129 (72)
Hospital	49 (28)
Locale before ICU admission	
Emergency department	119 (67)
General ward	59 (33)
Positive culture	
At any site	104 (58)
Gram-positive only	33/104 (32)
Gram-negative only	55/104 (53)
Fungus only	4/104 (4)
Mixed	12/104 (12)
Of blood	67 (37)
Gram-positive only	24/67 (36)
Gram-negative only	36/67 (54)
Fungus only	2/67 (3)
Mixed	5/67 (7)
Time to initiation of antibiotic therapy, hour	0 (-5.4 - 1.4)
Appropriate antibiotics^a^	82/104 (79)
Organ failure^b^	
Respiratory	138 (78)
Coagulation	109 (61)
Liver	64 (36)
Renal	67 (38)
Amount of fluid administered before vasopressor, L	1.6 (1.0-2.2)
Vasopressor (norepinephrine or equivalent) dose, μg/kg/min	0.48 (0.29 - 0.80)
Need for mechanical ventilation	124 (70)
Need for renal replacement therapy	58 (33)
CIRCI^c^	78/96 (81)
Relative adrenal insufficiency^c^	71/96 (74)
Time to initiation of low-dose corticosteroid therapy, hour	8.5 (3.8-19.1)

CIRCI, critical-illness-related corticosteroid insufficiency; SAPS, Simplified Acute Physiology Score; SOFA, Sequential Organ Failure Assessment. ^a^Appropriate antibiotics were based on the site of infection and available cultures.; ^b^Organ failure was defined as SOFA score of 2 or more per system; ^c^Results of adrenocorticotropic hormone test were available for 96 (54%) patients.

**Figure 1:**
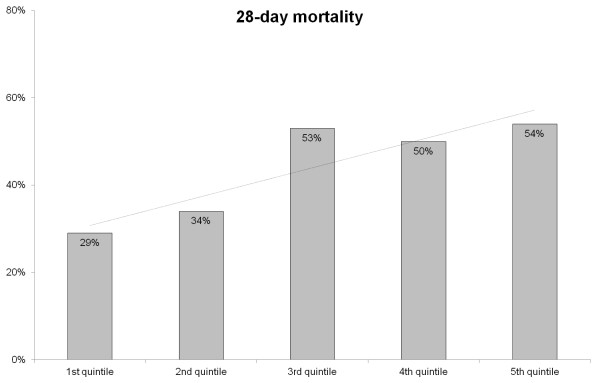
**Trend of 28-day mortality rate according to the time to initiation of low-dose corticosteroid therapy in quintiles (1^st ^quintile, 0 to 3.0 hours; 2^nd ^quintile, 3.1 to 6.3 hours; 3^rd ^quintile, 6.4 to 11.3 hours; 4^th ^quintile 11.4 to 24.5 hours; 5^th ^quintile, ≥ 24.6 h) (*P *= 0.0107, test for trend)**.

Reversal of shock was achieved in 67% after a median time of 35 (18 to 65) hours of low-dose corticosteroid therapy. Twenty eight-day mortality was 44%. ICU mortality was 43%, and median length of ICU stay was 6 (3 to 11) days. In-hospital mortality was 58%, and median length of hospital stay was 16 (7 to 31) days. Ninety-day mortality was 50%. Twenty eight-day mortality rates according to the time to initiation of low-dose corticosteroid therapy in quintiles are presented in Figure 1. The mortality rates increased significantly with increasing quintiles of time to initiation of low-dose corticosteroid therapy (*P *= 0.0107, test for trend).

Univariate comparisons of baseline characteristics at the time of initiation of low-dose corticosteroid therapy between survivors and non-survivors are presented in Table 2. There were no significant differences in age, sex, sites of infection, bacteremia, time to initiation of antibiotic therapy, or appropriate antibiotic treatment between groups. However, mortality was associated with severity of illness and organ failure requiring mechanical support. Median SAPS 3 and SOFA scores were significantly higher in non-survivors than in survivors (*P *< 0.0001 and *P *= 0.0007, respectively). Median dose of vasopressor at the time of initiation of low-dose corticosteroid therapy was higher in non-survivors compared with survivors (*P *< 0.0001). The need for mechanical ventilation and renal replacement therapy were also greater in non-survivors compared with survivors (*P *= 0.0001 and *P *< 0.0001, respectively). The median time to initiation of low-dose corticosteroid therapy was significantly shorter in survivors (6.5 hours, IQR 3.6 to 13.9 hours) than in non-survivors (10.4 hours, IQR 5.5 to 23.5 hours) (*P *= 0.0135). Although there was no difference in prevalence of CIRCI (81% in survivors versus 82% in non-survivors, *P *= 0.8679), the proportion of patients who underwent a reversal of shock within 48 hours after initiation of low-dose corticosteroid therapy was higher in survivors (74% versus 22%, respectively, *P *< 0.0001).

**Table 2:** Univariate comparisons of baseline characteristics at the time of initiation of low-dose corticosteroid therapy between survivors and non-survivors at 28 days

Variables	Survivors (number = 100)	Non-survivors (number = 78)	*P*-value
Age, years	63 (54-70)	68 (56-74)	0.1115
Sex, male	59 (59)	48 (62)	0.7315
Severity of illness			
SAPS 3	76 (69-87)	85 (81-95)	< 0.0001
SOFA	10 (8-12)	12 (10-15)	0.0007
Site of infection			0.5389
Lung	44 (44)	36 (46)	
Gastrointestinal tract	24 (24)	23 (30)	
Urinary tract	14 (14)	5 (6)	
Catheter related	0	5 (6)	
Skin and soft tissue	4 (4)	2 (3)	
Others	14 (14)	7 (9)	
Bacteremia	35 (34)	32 (41)	0.4103
Time to initiation of antibiotic therapy, hour	-0.2 (-5.2-1.3)	-0.4 (-3.1-2.1)	0.3977
Appropriate antibiotics^a^	52/61 (85)	30/43 (70)	0.057
Laboratory data			
Leukocytes, 10^3^/mm^3^	8.8 (1.6-19.9)	7.9 (0.3-17.0)	0.2221
Platelet, 10^3^/mm^3^	88 (43-179)	65 (35-102)	0.0235
Total bilirubin, mg/dL	1.4 (0.8-2.5)	1.4 (0.9-3.7)	0.3359
Creatinine, mg/dL	1.3 (0.9-2.1)	1.7 (1.0-2.9)	0.1326
Prothrombin time, %	61 (47-68)	53 (41-65)	0.058
Albumin	2.9 (2.5-3.3)	2.6 (2.3-3.0)	0.0083
Initial lactate, mmol/L	3.7 (2.6-5.5)	4.3 (2.9-9.6)	0.0382
Organ failure^b^			
Respiratory	71 (71)	67 (86)	0.0181
Coagulation	51 (51)	58 (74)	0.0015
Liver	34 (34)	30 (39)	0.5383
Renal	29 (29)	38 (49)	0.0071
Amount of fluid administered before vasopressor, L	1.5 (1.0-2.3)	1.6 (1.1-2.2)	0.263
Vasopressor (norepinephrine or equivalent) dose, μg/kg/min	0.36 (0.19-0.61)	0.67 (0.43-1.16)	< 0.0001
Need for mechanical ventilation	58 (58)	66 (85)	0.0001
Need for renal replacement therapy	18 (18)	40 (51)	< 0.0001
CIRCI^c^	46/57 (81)	32/39 (82)	0.8679
Relative adrenal insufficiency^c^	41/57 (72)	30/39 (77)	0.584
Time to initiation of low-dose corticosteroid therapy, hour	6.5 (3.6-13.9)	10.4 (5.5-23.3)	0.0135

95% CI, 95% confidence interval; CIRCI, critical-illness-related corticosteroid insufficiency; HR, hazard ratio; SAPS, Simplified Acute Physiology Score; SOFA, Sequential Organ Failure Assessment. ^a^Appropriate antibiotics were based on the site of infection and available cultures. ^b^Organ failure was defined as SOFA score ≥ 2 per system. ^c^The results of adrenocorticotropic hormone test were available for 96 (54%) patients.

The results of multivariate analyses with the logistic regression model are presented in Table 3. Time to initiation of low-dose corticosteroid therapy was associated with crude 28-day mortality (OR 1.015, 95% CI 1.001 to 1.030, *P *= 0.0483). After adjusting for *a priori *variables of age and gender (model 1), and time to initiation of antibiotic therapy and severity of illness (model 2), the association remained significant. The final logistic regression model (model 3) included the *a priori *parameters of age, gender, time to initiation of antibiotic therapy, and severity of illness assessed by SAPS 3 as well as other variables with *P*-values less than 0.25 on univariate analysis (Table 2). After adjusting for potential confounding factors, the time to initiation of low-dose corticosteroid therapy was still significantly associated with 28-day mortality (adjusted OR 1.025, 95% CI 1.007 to 1.044, *P *= 0.0075). Other factors independently associated with in-hospital mortality were higher SAPS 3 (adjusted OR 1.063, 95% CI 1.029 to 1.098, *P *= 0.0003), higher dose of vasopressor (norepinephrine or equivalent) (adjusted OR 1.921, 95% CI 1.014 to 3.639, *P *= 0.0453) and need for renal replacement therapy (adjusted OR 3.623, 95% CI 1.635 to 8.026, *P *= 0.0015).

**Table 3:** Associations between the time to initiation of low-dose corticosteroid therapy and 28-day mortality after adjustments for potential confounding factors

Time to initiation of low-dose corticosteroid therapy (hour)	Variables in the equation				
	
	Coefficient	SE	*P*-value	OR	95% CI
Crude state	0.0147	0.0074	0.0483	1.015	1.001-1.030
Adjusted state^a^					
Model 1	0.0147	0.0074	0.0483	1.015	1.001-1.030
Model 2	0.0239	0.0088	0.0065	1.024	1.007-1.042
Model 3	0.0249	0.0093	0.0075	1.025	1.007-1.044

95% CI, 95% confidence interval; OR, odds ratio; SE, standard error. ^a^Model 1 was adjusted for age and gender. Model 2 was additionally adjusted for SAPS 3 and time to initiation of antibiotic therapy. Model 3 additionally included leukocytes, platelet, creatinine, prothrombin time, albumin, initial lactate, vasopressor dose, the need for mechanical ventilation and continuous renal replace therapy.

**Figure 2:**
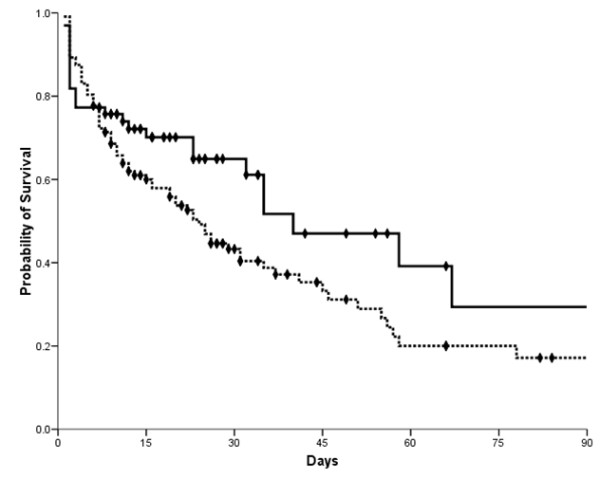
**Kaplan-Meier survival analysis comparing patients treated with low-dose corticosteroid therapy within six hours after development of septic shock and those treated later (solid line represents the early therapy group, who received the therapy within six hours; dotted line represents the late therapy group, who received the therapy after six hours)**.

Additionally, patients were categorized as early therapy group (low-dose corticosteroid administered within 6 hours from the initial onset of septic shock-related hypotension, *n *= 66) and late therapy group (low-dose corticosteroid administered after 6 hours from the initial onset of septic shock-related hypotension, *n *= 112). Both groups had similar characteristics, such as age, sex, severity of illness, sites of infection, bacteremia, time to initiation of antibiotic therapy, appropriate antibiotic treatment, laboratory data, and organ failure, except for need of mechanical ventilation (*P *= 0.0071) (Table 4). Although the proportion of patients who showed reversal of shock rate was similar in both groups (*P *= 0.0683), ICU mortality rate was better in the early-therapy group (32%) than in the late-therapy group (49%) (*P *= 0.0243). Finally, the early therapy group had a 37% lower 28-day mortality rate than the late therapy group (32% versus 51%, *P *= 0.0132) and a similar difference in mortality rate was observed up to 90 days (Table 4 and Figure 2). After adjusting for potential confounding factors, initiation of low-dose corticosteroid therapy more than 6 hours after the onset of septic shock was independently associated with increased 28-day mortality (adjusted OR 2.142, 95% CI 1.047 to 4.382, *P *= 0.0369) (Table 5).

**Table 4:** Baseline characteristics, therapy, and outcomes between early (≤ 6 hour) and late (> 6 hour) low-dose corticosteroid therapy groups

Variables	Early group, ≤ 6 hour (number = 66)	Late group, > 6 hour (number = 112)	*P*-value
Age, years	67 (55-71)	65 (53-71)	0.8142
Sex, male	42 (64)	65 (58)	0.4611
Severity of illness			
SAPS 3	80 (71-89)	82 (72-91)	0.146
SOFA	11 (9-13)	11 (9-14)	0.6498
Site of infection			0.7997
Lung	27 (41)	53 (47)	
Gastrointestinal tract	20 (30)	27 (24)	
Urinary tract	9 (14)	10 (9)	
Catheter related	3 (4)	2 (2)	
Skin and soft tissue	1 (2)	5 (4)	
Others	6 (9)	15 (14)	
Bacteremia	28 (42)	39 (35)	0.3119
Time to initiation of antibiotic therapy, hour	0.1 (-3.3-1.2)	0.1 (-4.9-1.9)	0.9659
Appropriate antibiotics^a^	36/42 (86)	46/62 (74)	0.1581
Laboratory data			
Leukocytes, 10^3^/mm^3^	6.9 (0.3-16.5)	10.2 (0.1-18.1)	0.1797
Platelet, 10^3^/mm^3^	75 (35-148)	68 (40-155)	0.5662
Total bilirubin, mg/dL	1.4 (0.9-2.7)	1.4 (0.9-1.7)	0.9519
Creatinine, mg/dL	1.7 (1.0-2.5)	1.4 (0.9-2.4)	0.1673
Prothrombin time, s	61 (47-68)	54 (42-66)	0.2931
Albumin, g/dL	3.0 (2.3-3.3)	2.6 (2.4-3.1)	0.0811
Initial lactate, mmol/L	4.1 (2.8-5.7)	3.8 (2.6-6.6)	0.8798
Organ failure^b^			
Respiratory	48 (73)	90 (80)	0.2388
Coagulation	43 (65)	66 (59)	0.4105
Liver	24 (36)	40 (36)	0.9305
Renal	28 (42)	39 (35)	0.3119
Amount of fluid administered before vasopressor, L	1.6 (1.0-2.3)	1.5 (1.1-2.2)	0.1932
Vasopressor (norepinephrine or equivalent) dose, μg/kg/min	0.50 (0.32-1.06)	0.52 (0.29-0.80)	0.6277
Need for mechanical ventilation	38 (58)	86 (77)	0.0071
Need for renal replacement therapy	19 (29)	39 (35)	0.4068
CIRCI^c^	16/23 (70)	62/73 (85)	0.1269
Relative adrenal insufficiency^c^	15/23 (65)	56/73 (77)	0.2733
Outcomes			
Reversal of shock	50 (76)	70 (63)	0.0683
ICU mortality	21 (32)	55 (49)	0.0243
Length of stay in ICU, days	4 (3-8)	7 (4-12)	0.004
28-day mortality	21 (32)	57 (51)	0.0132
90-day mortality	27 (41)	72 (64)	0.0024
In-hospital mortality	29 (44)	75 (67)	0.0026
Length of stay in hospital, days	16 (7-28)	18 (7-30)	0.6974

CIRCI, critical-illness-related corticosteroid insufficiency; SAPS, Simplified Acute Physiology Score; SOFA, Sequential Organ Failure Assessment. ^a^Appropriate antibiotics were based on the site of infection and available cultures. ^b^Organ failure was defined as SOFA score of ≥ 2 or more per system. ^c^The results of adrenocorticotropic hormone test were available for 96 (54%) patients.

**Table 5:** Multivariate analysis with forward stepwise multiple logistic regression model for probability of 28-day mortality in patients receiving low-dose corticosteroid therapy

Variables	Adjusted OR	95% CI	*P*-value
SAPS 3	1.059	1.028-1.091	0.0001
Need for renal replacement therapy	4.191	2.026-8.668	0.0369
Late (> 6 hour) low-dose corticosteroid therapy	2.142	1.047-4.382	0.0369

95% CI, 95% confidence interval; OR, odds ratio; SAPS, Simplified Acute Physiology Score.

## Discussion

This is the first study to evaluate the association between early initiation of low-dose corticosteroid therapy and decreased mortality in patients with septic shock. The results of our retrospective cohort study indicate that early administration of low-dose corticosteroid is independently associated with better outcome in patients with septic shock. Additionally, we found that early low-dose corticosteroid therapy within 6 hours from the initial onset of septic shock-related hypotension reduced the relative risk of 28-day mortality by 37% compared with late therapy after 6 hours.

All patients with septic shock in our study received low-dose corticosteroid therapy as a standard treatment protocol. However, time to initiation of this therapy depended on the decision of the primary physician. Therefore, we were able to analyze the association between the time to initiation of low-dose corticosteroid therapy and outcomes. Our findings could partially explain the different results of two large multicenter, randomized, controlled studies of low-dose corticosteroid therapy, which used different time windows of enrollment [9,10]. In the study by Annane *et al*., in which low-dose corticosteroid therapy was initiated within eight hours after the onset of septic shock, a survival benefit was seen in patients with no response to corticotropin [9]. In that study, mean time from initiation of vasopressor to study drug therapy was about four hours. In contrast, because this time window was subsequently increased to 72 hours after the onset of septic shock in the CORTICUS study, the benefit of this therapy was not shown [10]. Although 77% of enrolled patients were started on the study treatment within 12 hours after the onset of septic shock in the CORTICUS study, there were no significant differences in outcome in this subgroup [10,22]. In the present study, the median time to initiation of low-dose corticosteroid therapy was 8.5 hours after the onset of septic shock, and early administration of low-dose corticosteroid was independently associated with decreased mortality even after adjustment for potential confounding factors, including severity of illness, which was another important difference between the two previous large studies. Specifically, when the outcomes were compared between patients treated within six hours after development of septic shock and those treated later, baseline characteristics including severity of illness were not different, but a better outcome was observed in patients receiving low-dose corticosteroid therapy within six hours.

Corticosteroids have potent anti-inflammatory effects and were the first drugs tested in large randomized controlled trials in septic patients [23-25]. However, these studies indicated that a short course of high-dose corticosteroid in early stages of septic shock had no effect on outcome or was even harmful, probably because of immunosuppression and increased incidence of secondary infections [5-7]. Only one study showed an initial improvement in survival and shock reversal with high-dose corticosteroid, but with ongoing disease, the difference was no longer significant [23]. Recently, the observation that severe sepsis and septic shock may be associated with absolute or relative adrenal insufficiency and steroid receptor resistance prompted renewed interest in steroid treatment in septic patients [26,27]. A novel approach of low-dose corticosteroid was found to have beneficial effects on hemodynamics and outcome [7-9]. These effects have mainly been attributed to sensitization of the vasculature to vasopressor [28]. However, there is other evidence that this low-dose corticosteroid therapy affects the immune response [29-31]. Briegel *et al*. [29] reported that low-dose corticosteroid therapy was associated with significant reductions in proinflammatory cytokine levels and with early resolution of sepsis-induced organ dysfunction. Keh *et al*. [30] also demonstrated that low-dose corticosteroid therapy inhibited systemic inflammation and prevented overwhelming compensatory anti-inflammatory responses. Moreover, in a recent randomized controlled study, Oppert *et al*. [31] found that the anti-inflammatory effects of low-dose corticosteroid were independent of adrenal function. Therefore, if the benefits of low-dose corticosteroid therapy in the management of septic shock are not limited to hemodynamic improvement in patients with impaired adrenal functions but are also related to anti-inflammatory effects, timely initiation of this treatment would be important. Unfortunately, our study has no data related to the anti-inflammatory effects of low-dose corticosteroid therapy.

Several reports provided additional evidence of the importance of timely intervention in the management of septic shock. Rivers *et al*. [14] reported that early hemodynamic resuscitation (EGDT) for severe sepsis and septic shock within six hours resulted in improved survival compared with less prompt resuscitation treatment. Kumar *et al*. [17] showed that from the onset of hypotension in patients with septic shock, each hour of delay in antibiotic therapy over the ensuing six hours was associated with an increase in mortality. In the present study, initiation of low-dose corticosteroid therapy more than six hours after the onset of septic shock was independently associated with increased mortality after adjusting for potential confounding factors.

To fully appreciate these results, the limitations of this study must be acknowledged. First, given its retrospective nature, there is always the possibility that selection bias may have influenced the significance of our findings. Previous observational studies reported that patients treated with low-dose corticosteroids are more severely ill [32,33]. Therefore, more severely ill patients might receive delayed initiation of low-dose corticosteroid therapy compared to patients who received early therapy in this study. However, baseline severity or number of organ failures at the time of initiation of low-dose corticosteroid therapy was not different between the early treatment group and the late treatment group. In addition, adjusted multivariate analysis, and the protocolization of initial resuscitation and management for severe sepsis and septic shock before the initiation of this study served to minimize the potential for selection bias. However, the potential for a bias of unmeasured confounder remains. Second, the data on initial resuscitation could not be extracted due to incomplete medical records. Compliance with initial resuscitation bundles and achievement of end points may be important confounding factors in the association between time to initiation of low-dose corticosteroid therapy and mortality. Other data of our EAST program on the initial resuscitation for severe sepsis and septic shock in the emergency department for one year (from August, 2008 to July, 2009) noted that compliance of resuscitation bundle was 50% (unpublished data). However, it is not clear how compliance with EGDT and achievement of end points would affect this association. Furthermore, there are potential barriers to the effective implementation of EGDT at the patient, clinician, and organizational level [34]. Therefore, our results represent actual practice at a tertiary referral hospital. Third, the reason for delayed initiation of low-dose corticosteroid therapy could not be evaluated.

## Conclusions

The results of this study demonstrated a significant association between early initiation of low-dose corticosteroid therapy and decreased mortality rate in patients with septic shock. However, this observation needs further evaluation with a prospective, randomized, controlled study.

## Key messages

• Early initiation of low-dose corticosteroid therapy is significantly associated with decreased mortality in patients with septic shock.

• Additionally, early low-dose corticosteroid therapy within 6 hours from the initial onset of septic shock-related hypotension reduced the relative risk of 28-day mortality by 37% compared with late therapy after 6 hours from the hypotension.

## Abbreviations

ACTH: adrenocorticotropic hormone; CI: confidence intervals; CIRCI: critical-illness-related corticosteroid insufficiency; CVP: central venous pressure; EGDT: early goal-directed therapy; HR: hazard ratios; IQR: interquartile range; MAP: mean arterial pressure; SAP 3: Simplified Acute Physiology Score 3; ScvO_2_: central venous oxygen saturation; SOFA: Sequential Organ Failure Assessment.

## Competing interests

The authors declare that they have no competing interests.

## Authors' contributions

HYP collected and analyzed the data and drafted this manuscript. GYS contributed to the design of this study, analysis of the data, and writing of the manuscript. JS collected data and assisted with analyzing the data and drafting the manuscript. HY collected data and assisted with analyzing the data and revising the manuscript. IJJ contributed to analysis and interpretation of data and revising the manuscript. TGS collected data and assisted with analyzing the data and drafting the manuscript. SYL collected data and assisted with analyzing the data and drafting the manuscript. SW conducted statistical analyses and interpreted the data. KJ conceived and designed the study, analyzed the data, and wrote the final manuscript. All authors have read and approved the final manuscript.
